# Correction: Dietary adherence among persons with type 2 diabetes: A concurrent mixed methods study

**DOI:** 10.1371/journal.pone.0344783

**Published:** 2026-03-10

**Authors:** Dorothy Wilson, Abigail Kusi-Amponsah Diji, Richard Marfo, Paulina Amoh, Precious Adade Duodu, Samuel Akyirem, Douglas Gyamfi, Hayford Asare, Jerry Armah, Nancy Innocentia Ebu Enyan, Joana Kyei-Dompim

In the Results section of the Abstract, there is an error in the second sentence. The correct sentence is: In multivariable logistic regression, it was found that increase in DRNK (AOR = 1.101, 95% CI: 1.004–1.208, p = 0.041) and living in an urban area (AOR = 3.058, 95% CI: 1.014–9.221, p = 0.047) were significantly associated with good dietary adherence.

Reference 2 is incorrect. The correct reference is: Waxman A, World Health Assembly. WHO global strategy on diet, physical activity and health. Food Nutr Bull. 2004 Sep;25(3):292–302.

There is an error in reference 27. The correct reference is: Kroll T, Neri M. Designs for mixed methods research. In: Andrew S, Halcomb EJ, editors. Mixed Methods Research for Nursing and the Health Sciences. 2009. p. 31–49.
