# Supplementary material for: Dietary adherence among persons with type 2 diabetes: A concurrent mixed methods study
Source: PLoS One. 2024 May 7;19(5):e0302914. doi: 10.1371/journal.pone.0302914 (PMC11075895; doi:10.1371/journal.pone.0302914)
Supplement: S1 File — (DOCX) [file pone.0302914.s001.docx]

**Questionnaire for participants**

**Section A**

| **Sociodemographic characteristics**    (**Instruction**: Please complete this section by ticking the appropriate answers or writing as required) | |
| --- | --- |
| Age | ………………………… |
| Sex | Male Female |
| Marital status | Single Married Divorced  Separated |
| Place of residence | ……………………… |
| Educational Status | Primary Education Secondary Education Tertiary Education None |
| Employment | Private sector employment Public sector employment  Self-employment No employment |
| Monthly income (in Ghana Cedis) | <500 500-900 1000-2000  >2000 |

| **Clinical data**  (Instruction: Please complete this section by ticking the appropriate answers or writing as required) | |
| --- | --- |
| Family history of diabetes mellitus | Yes No |
| Comorbidity | Yes No |
| Duration of condition | Less than 5 years  5 years and above |

**Section B**

**Assessment of dietary adherence**

(**Instruction**: please circle one option from the response section)

| ITEMS | RESPONSE |
| --- | --- |
| 1. On how many of the last SEVEN DAYS have you followed dietary recommendations given by your healthcare provider? | 0 1 2 3 4 5 6 7 |
| 1. On how many of the last SEVEN DAYS did you eat the number of fruit and vegetables you are supposed to eat based on recommendations from your healthcare provider? | 0 1 2 3 4 5 6 7 |
| 1. On how many of the last SEVEN DAYS did you eat carbohydrate-containing foods with a low Glycaemic Index? (Example: beans, oatmeal, pasta, low fat dairy products) | 0 1 2 3 4 5 6 7 |
| 1. On how many of the last SEVEN DAYS did you eat foods high in sugar, such as cakes, cookies, desserts, candies, etc.? | 0 1 2 3 4 5 6 7 |
| 1. On how many of the last SEVEN DAYS did you eat foods high in fibre such as oatmeal, high fibre cereals (wheat, brown rice), and whole-grain breads? | 0 1 2 3 4 5 6 7 |
| 1. On how many of the last SEVEN DAYS did you space carbohydrates evenly throughout the day? | 0 1 2 3 4 5 6 7 |
| 1. On how many of the last SEVEN DAYS did you eat fish or other foods high in omega-3(shrimp, soybean oil)? | 0 1 2 3 4 5 6 7 |
| 1. On how many of the last SEVEN DAYS did you eat foods that contained or was prepared with sunflower, soybean or olive oils? | 0 1 2 3 4 5 6 7 |
| 1. On how many of the last SEVEN DAYS did you eat foods high in fat (such as high fat dairy products, fatty meat, fried foods or deep-fried foods)? | 0 1 2 3 4 5 6 7 |

**Section C**

**Assessment of diabetes-related nutrition knowledge**

(**Instruction:** Please complete this section by ticking the appropriate answers)

1. Does healthcare professional recommend that persons living with type 2 diabetes should be eating more, the same amount or less of the following foods? (Tick one box per food)

More Same Less Not sure

Fruits

Food and drinks with added sugar

Vegetables

Fatty foods

Processed red meat

Wholegrains

1. How many servings of fruit and vegetables per day do healthcare professional advice persons living with type 2 diabetes to eat as a minimum? (One serving could be, for example and apple or a handful of chopped carrots) (Tick one)

2

3

4

5 or more

Not sure

1. Which one of these types of fats do healthcare professional recommend that persons living with type 2 diabetes should eat less of? (Tick one box per food)

Eat less Not eat less Not sure

Olive oil, sunflower oil, soybean oil, etc.

Margarine, peanut butter, French fries, etc.

Fatty meat (like pork), coconut oil, cheese

1. Do you think these foods are typically high or low in fiber? (Tick one box per food)

High in fiber Low in fiber Not sure

Oats

Banana

White rice

Eggs

Pasta

1. Which of the following foods do experts count as starchy foods? (Tick one box per food)

Starchy food Not starchy Not sure

Pasta

Potatoes

Nuts

Plantain

1. Which would be the healthiest and most balanced choice for a main meal in a restaurant? (Tick one)

Locally cooked rice with lots of vegetables and roasted chicken

Fired rice made with polished rice and beef with cream

Potato chipped served with chicken and tomato ketchup

Not sure

1. Do you think these foods and drinks are typically high or low in added sugar? (Tick one box per food)

High in added sugar Low in added sugar Not sure

Diet cola drinks

Natural yogurt

Ice cream

Tomato ketchup

Melon

1. Which one of these foods is more likely to raise people’s blood cholesterol? (Tick one)

Eggs

Vegetable oil

Animal fats

Not sure

1. Which one of these foods is classified as having a high Glycaemic Index (Glycaemic Index is a measure of the impact of a food on blood sugar levels, thus a high Glycaemic Index means a greater rise in blood sugar after eating)? (Tick one)

Wholegrain cereals

White bread

Fruits and vegetables

Not sure
