# Supplementary material for: Dietary adherence among persons with type 2 diabetes: A concurrent mixed methods study
Source: PLoS One. 2024 May 7;19(5):e0302914. doi: 10.1371/journal.pone.0302914 (PMC11075895; doi:10.1371/journal.pone.0302914)
Supplement: S2 File — (DOCX) [file pone.0302914.s002.docx]

**DIETARY ADHERENCE AMONG PERSONS**

**WITH TYPE 2 DIABETES: A MIXED METHODS STUDY**

**INTERVIEW GUIDE**

**Purpose**: It is designed to explore the perspective of persons with type 2 diabetes on the facilitators and barriers to adherence to dietary recommendations.

**Introduction:**

We are a team of researchers from the Department of Nursing, Kwame Nkrumah University of Science and Technology (KNUST). We are conducting research into exploring the factors that influence dietary adherence among persons with type 2 diabetes.

We would like to know your perspectives and experiences with regard to the facilitators and barriers to dietary adherence. We would be very appreciative of anytime spent sharing your valuable information on this situation with us. This interview is expected to take a maximum of 30 minutes of your time. Be assured of confidentiality of the information you will provide for this research.

**Biodata**

Participant Study ID…….

1. Can you please tell us a bit about yourself?
2. Age
3. Place of residence
4. Marital status
5. Highest level of education
6. Employment status
7. monthly income

**Main questions**

1. Have you received any dietary recommendations from healthcare professionals since your diagnosis?
2. Are you able to adhere to these recommendations?
3. What factors have enabled you to adhere to these recommendations?
4. What factors have made it challenging for you to adhere to the dietary recommendations received from health professionals?
