# Supplementary material for: Dietary adherence among persons with type 2 diabetes: A concurrent mixed methods study
Source: PLoS One. 2024 May 7;19(5):e0302914. doi: 10.1371/journal.pone.0302914 (PMC11075895; doi:10.1371/journal.pone.0302914)
Supplement: Attachment — Submitted filename: Peer review Plos One.docx [file pone.0302914.s004.docx]

The study investigates dietary adherence among individuals with Type 2 diabetes in Ghana. Its primary objectives are to identify predictors of dietary adherence and explore the influencing factors. To achieve these goals, the study adopts a mixed-methods approach, combining quantitative surveys and qualitative interviews. The study's findings reveal that approximately half of the participants exhibit good dietary adherence. Various factors impact adherence, including diabetes-related nutrition knowledge, financial challenges, social environments, individual food preferences, and access to healthy food choices.

**Suggestions for Improvement:**

1. The authors should present in the introduction the main findings of studies done in Ghana with quantitative and qualitative data alone. This would provide important context for readers and help them understand the study's significance within the existing body of research.
2. The authors should explain why triangulation is a novel and superior approach compared to quantitative or qualitative studies alone, providing an example. This explanation can help readers understand the rationale behind the chosen research methodology and its advantages.
3. The authors should provide more detailed information about the sampling strategy, participant selection, randomization, and how the sample represents the target population.
4. The authors should offer insights into the characteristics of non-participants and discuss potential sources of bias introduced by non-participation, enhancing transparency in the study.
5. The authors should describe validity and reliability of the tools and instruments used for data collection, including survey instruments. This information helps readers assess validity of the study's findings.
6. The authors should offer a brief outline of the statistical methods used for analyzing quantitative data, specifying types of tests and significance levels for clarity.
7. The authors should provide a concise description of the qualitative data analysis process, including theme derivation, coding, and software tools used, ensuring transparency in qualitative analysis.
8. The authors should consider merging quantitative results from Tables 3 and 4 into a single regression analysis, addressing collinearity issues for streamlined presentation.
9. The authors should provide a more comprehensive and critical comparison of the study's findings with existing literature, exploring both similarities and differences and discussing implications for the field's understanding. This in-depth comparison helps readers contextualize the study's contributions and assess its significance in the broader research landscape.
