# Supplementary material for: Dietary adherence among persons with type 2 diabetes: A concurrent mixed methods study
Source: PLoS One. 2024 May 7;19(5):e0302914. doi: 10.1371/journal.pone.0302914 (PMC11075895; doi:10.1371/journal.pone.0302914)
Supplement: Attachment — Submitted filename: Response to Reviewers.docx [file pone.0302914.s005.docx]

**RESPONSE TO REVIEW COMMENTS ON MANUSCRIPT WITH REFERENCE NUMBER: PONE-D-23-08604**

First, we would like to thank the editor and reviewers for their valuable comments and suggestions. We have carefully considered their feedback and revised the manuscript accordingly. Please find in the table below the responses to the review comments.

| **ADDITIONAL EDITOR’S COMMENTS** | |
| --- | --- |
| **COMMENT RESPONSE** | |
| 1. Please ensure that your manuscript meets PLOS ONE's style requirements, including those for file naming. | Thank you for the comment. We have prepared the manuscript based on the PLOS ONE style requirements. |
| 2. Please include captions for your Supporting Information files at the end of your manuscript, and update any in-text citations to match accordingly. | Thank you for the comment. We have included captions for Supporting Information files at the end of the manuscript. Please refer to page 52, lines 621 to 624. In-text citations have also been updated. |
| **REVIEWER 1 COMMENTS** | |
| The authors report their findings from a mixed methods study on adherence to dietary recommendations among persons living with diabetes in urban settings in Ghana . Their paper is generally well written with few language use errors which need to be corrected. Review of the paper by a native English speaker would be beneficial. | Thank you for pointing this out. The manuscript has been reviewed by a native English speaker and the language errors have been corrected. |
| The methods, results and discussion are congruent however the study design chosen is inappropriate for the aim. A longitudinal design is required for determining predictors. At best a cross-sectional design may yield associations. | Thank you for the comment. We agree with this comment. We have accordingly revised the study’s aim. The revised aim is to identify the factors associated with dietary adherence and explore the barriers and faciltators to dietary adherence. Please refer to page 2, lines 17 and 18 and page 8, line 107 and 108. |
| The authors use p-values in deciding on the variable selection when modelling. Data-driven selection of variables is a methodological no go and known to result in spurious associations. Selection of variables should rather be based on theoretical knowledge and biological plausibility. The authors should consider this and re-run the qualitative analysis. | Thank you for your comments. Based on your recommendation we have re-run the quantitative analysis. The variables included in the model were selected based on evidence from previous literature conducted in Ghana and other countries; on the factars associated with dietary adherence. Please refer to page 14, lines 202 to 204 and to pages 24 to 27, Table 3 for the results of the analysis.  However, we believe that the qualitative analysis was well conducted and the results reflect the views of the participants. Additionally, your preceding comments are related to quantitative analysis rather than qualitative analysis. |
| **REVIEWER 2 COMMMENTS** | |
| 1. The authors should present in the introduction the main findings of studies done in Ghana with quantitative and qualitative data alone. This would provide important context for readers and help them understand the study's significance within the existing body of research. | Thank you for the suggestion. This has been included in the introduction. Please refer to pages 6 and 7, lines 78 to 92. |
| 2. The authors should explain why triangulation is a novel and superior approach compared to quantitative or qualitative studies alone, providing an example. This explanation can help readers understand the rationale behind the chosen research methodology and its advantages | Thanks for the comment. This has been included in the introduction. Please refer to pages 7 and 8, line 92 to 106. |
| 3. The authors should provide more detailed information about the sampling strategy, participant selection, randomization, and how the sample represents the target population | Thanks for the comment. We have accordingly added more information to emphasize this point. For the quantitative sampling, we explained how we used consecutive sampling to select participants who met the inclusion criteria until the desired sample size was reached. Please refer to page 10, line 134. Also for the qualitative phase, we explained how researchers selected participants from the quantitative sample during data collection for the quantitative phase. We purposefully selected participants we believed would provide valuable information. These judgements were made based on researchers’ interaction with participants during the quantitative phase. Please refer to page 10, lines 137 to 141. |
| 4. The authors should offer insights into the characteristics of non-participants and discuss potential sources of bias introduced by non-participation, enhancing transparency in the study.   . | Thank you for pointing this out. We have incorporated your suggestion in the manuscript. The characteristics of non-participants based on the inclusion and exclusion criteria outlined in the manuscript have been provided in the limitations section of the manuscript. We added that the exclusion of patients with certain characteristics may be a source of bias and may limit the generalizability of the findings to all patients with type 2 diabetes in Ghana. However, we emphasized that the findings of the study are relevant for improving dietary adherence despite this limitation. Please refer to page 42, lines 486 to 490 and lines 493 to 497. |
| 5. The authors should describe validity and reliability of the tools and instruments used for data collection, including survey instruments. This information helps readers assess validity of the study's findings | Thank you for the comment. The validity and reliability of the tools used for quantitative data collection have been provided in the manuscript. The Cronbach Alpha of the questionnaires were reported as well as the process for establishing face and content validity. Please refer to page 16, lines 226 to 234.  Based on your comments, the process for establishing the content validity of the interview guide has been added. Please refer to page 16, lines 235 to 238. |
| 6. The authors should offer a brief outline of the statistical methods used for analyzing quantitative data, specifying types of tests and significance levels for clarity. | Thank you for the comment. The specific tests used for quantitative data analysis and the level of significance considered in the analysis have been reported in the manuscript. The reasons for using specific tests and the variables included were also outlined. The significance level was set at ≤0.05. Please refer to page 14, lines 199 to 210. |
| 7. The authors should provide a concise description of the qualitative data analysis process, including theme derivation, coding, and software tools used, ensuring transparency in qualitative analysis | Thank you for your suggestion. Details on the qualitative analysis have been added; proving information on the analysis process and the software tool used which is Nvivo software, version 20.0. |
| 8. The authors should consider merging quantitative results from Tables 3 and 4 into a single regression analysis, addressing collinearity issues for streamlined presentation | Thank you for the comment. The tables have been merged. The table contains results of the Spearman’s correlarion, chi-square test and binary logistic regression. Please refer to pages 24-27, Table 3. |
| 9. The authors should provide a more comprehensive and critical comparison of the study's findings with existing literature, exploring both similarities and differences and discussing implications for the field's understanding. This in-depth comparison helps readers contextualize the study's contributions and assess its significance in the broader research landscape. | Thank you for the comment. The discussion has been improved by critically comparing the study’s findings with others studies and establishing differences or similarities between quantitative and qualitative findings of the study. Please refer to pages of 38 and 39, lines 416 to 441; page 40; page 41, line 473. |
