# Supplementary material for: Dietary adherence among persons with type 2 diabetes: A concurrent mixed methods study
Source: PLoS One. 2024 May 7;19(5):e0302914. doi: 10.1371/journal.pone.0302914 (PMC11075895; doi:10.1371/journal.pone.0302914)
Supplement: Attachment — Submitted filename: Response to Reviewers.docx [file pone.0302914.s006.docx]

**RESPONSE TO REVIEW COMMENTS ON MANUSCRIPT WITH REFERENCE NUMBER: PONE-D-23-08604**

First, we would like to thank the editor and reviewers for their valuable comments and suggestions. We have carefully considered their feedback and revised the manuscript accordingly. Please find in the table below the responses to the review comments.

| **ADDITIONAL EDITOR’S COMMENTS** | |
| --- | --- |
| **COMMENTS** | **RESPONSES** |
| **1.Correct the in-text reference in page 6 (Doglikuu et al 2021). Be consistent with the referencing style.** | Thank you for pointing this out. We have ensured that all references are in Vancouver style. |
| **2. The facilities sampled from have a combined T2DM attendance of 200, why did you have to calculate a sample size. The easiest will be to utilize the entire sample and do a retrospective power calculation to confirm whether you are adequately powered. The approach for the sample calculation problematic and that is the main point of the second reviewer.** | Thank you for the comment. While we generally agree with the reviewer that recruiting the entire population of T2D individuals from the two facilities would have been ideal, we also understand that this was not feasible as some patients may decline participation or may not be reachable during the study period. The Yamane Taro approach to sample size calculation enabled us to determine the minimum number of participants that can be considered as representative of a finite population size of 200. That said, we have performed a retrospective power analysis with the 146-sample size and have provided details of this in the manuscript. Please refer to pages 9-10, lines 185 to 188. |
| 3. Add a section to your methods where you define all the variables used. For instance, how did you define rural and urban for patients attending two facilities within an urban municipality in the Ashanti region. | Thank you for the comment. A section for definition of independent and dependent variables has been included in the methods. Please refer to pages 16-17, lines 324 to 336.  However, the two health facilities are within two different municipalities in the Ashanti region. While the KNUST Hospital is in the Oforikrom municipal, which is an urban municipal, the Ejisu Government Hospital is in the Ejisu Municipal, which is made up of both urban and rural localities (based on the Ghana 2021 Population and Housing Census General Report Volume 3A: Population of Regions and Districts). This information has been added to the section for study setting in the methods and the names of the facilities have been included. Please refer to pages 7-8, lines 142 to 150. For the place of residence, the name of the locality of residence was obtained from the participants and were designated as rural or urban. All localities within the Oforikrom Municipality were designated as urban. For the Ejisu municipal, all localities apart from Ejisu (Krapa inclusive), Kwamo and Fumesua were designated as rural (based on information from the Ministry of Food and Agriculture website; https://mofa.gov.gh/site/sports/district-directorates/ashanti-region/162-ejisu-juaben and Openalfa Street Directories; https://ghana-streets.openalfa.com/ejisu-municipal-district). Please refer to page 11, lines 211 to 215. |
| 4. You indicate media scores were used for you PDAQ and DRNK yet in table 2 you present mean and standard deviation. Correct this to median scores with IQR for clarity to the reader. | Thank you for the comment. The mean and standard deviation in Table 2 have been changed to median and interquartile range respectively. Please refer to pages 21-23. |
| 5. Summarize and discuss the overlap (triangulation) of quantitative and qualitative findings/results. You could illustrate this on put in a table. | Thank you for the suggestion. The quantitative and qualitative findings have been integrated and presented in the results section using the joint display method as described by Fetters et al. in their article titled; “Achieving Integration in Mixed Methods Designs — Principles and Practices” (https://onlinelibrary.wiley.com/doi/10.1111/1475-6773.12117). Please refer to Table 5 on pages 36-43. |
| **REVIEWER 1 COMMENTS** | |
| **COMMENTS** | **RESPONSES** |
| 1. The authors indicate a modification in the study aim to study associations rather than predictors. This is possible with a cross-sectional design but this must be an analytical cross-sectional design . The sample size formula used is for one sample with finite correction. This is a fundamental flaw.The investigators need to demonstrate that the sample size is adequate to be able to estimate the population parameter in this case OR | Thank you for the comment. We have indicated in the manuscript that the quantitative phase of the study employed an analytical cross-sectional design. Please refer to page 7, lines 126 to 127.Concerning the sample size, we have performed a retrospective power analysis using GPower and we determined that a sample size of 146 will yield >99% power at 95% confidence level. This determination was based on an assumed odds ratio of 4.7 between diabetes knowledge and dietary adherence as reported in a previous study in Sudan (reference provided in manuscript) and a two-tailed z-test. Please refer to page 9-10, lines 185 to 188. |
| **REVIEWER 2 COMMENTS** | |
| **COMMENTS** | **RESPONSES** |
| 1.It appears that the manuscript is currently in landscape layout. If this is the case, I recommend changing it to portrait format for better readability. | Thank you for the suggestion. The manuscript layout has been changed to portrait. |
| 2.The introduction, while comprehensive, could benefit from being more concise for clarity. | Thank you for the suggestion. While we thought a comprehensive introduction could put the topic in context, we have attempted to make it a bit more concise. Please refer to pages 4-6 |
| 3. Please provide the baseline N for the sample size calculation | Thank you for the comment. The baseline N for the sample size was 200 and it has been indicated in the manuscript. Please refer to page 9, lines 181-182. |
| 4. Include the justification for conducting the study at the two hospitals. | Thank you for the suggestion. The study sought to target both rural and urban population. Hence, the choice of the KNUST Hospital and Ejisu Government Hospital is based on their diverse patient populations. The KNUST Hospital provides services to a mix of students, university staff and locals while the Ejisu Government Hospital serves both rural and urban communities.Please refer to page 8, lines 156-162. |
| 5.Clearly explain the rationale for selecting 14 participants and outline the criteria used to make this decision. | Thank you for the comment. Data saturation (when new data replicates what has already been gathered) was achieved after conducting 14 interviews. The researchers selected participants who differ across multiple criteria such as age, sex, place of residence, income, education level, employment, and marital status. Please refer to page10, lines 192 to 205. |
| 6. In the table presenting regressions, consider using the term "reference" instead of numerical values (1 or dashes) for clarity purposes. | Thank you for the suggestion. The numerical values (1 and dashes) have been deleted and the abbreviation ***ref*** (reference category)*,* has been used to indicate categorical variables used as reference categories in the regression analyses. Please refer to Table 3, pages 23 to 26. |
| 7. Additionally, I suggest consolidating all statistically significant factors into one logistic regression model as predictors. Exclude collinear variables using variance inflation factors, and then report the final model, explicitly revealing the most important determinants | Thank you for the comment. However, the primary purpose of the study is not to determine predictors of dietary adherence but to identify factors associated with dietary adherence. To determine predictors, a longitudinal study design will be required. |
